# ENSO modulates aerobic habitat across varying hypoxia tolerance levels in the Southeast Pacific throughout the twenty-first century

**DOI:** 10.1038/s41598-025-06498-5

**Published:** 2025-07-01

**Authors:** A. Parouffe, B. Dewitte, A. Paulmier, V. Garçon

**Affiliations:** 1 Laboratoire d’Etudes en Géophysique et Océanographie Spatiales, LEGOS (CNES/CNRS/IRD/UPS), Université de Toulouse, Toulouse, France; 2 Centro de Estudios Avanzados en Zonas Aridas (CEAZA), Coquimbo, Chile; 3 CECI, CERFACS/CNRS, Université de Toulouse, Toulouse, France; 4 Departamento de Biología, Facultad de Ciencias del Mar, Universidad Católica del Norte, Coquimbo, Chile; 5 CNRS/IPGP, Institut de Physique du Globe de Paris, Paris, France

## Abstract

The ENSO (El Niño Southern Oscillation), the planet’s most consequential climate mode, imposes significant thermal stress on epipelagic marine ecosystems. However, its effects on aerobic habitats within the epipelagic and mesopelagic zones remain largely uncharted. This study examines these impacts in the Southeast Pacific, a region hosting one of the world’s most pronounced Oxygen Minimum Zones (OMZ), focusing on species with varying hypoxia tolerances. Using Earth System Model simulations, we show that key characteristics of ENSO—its amplitude, spatial and temporal asymmetry referred to as ENSO diversity—significantly affect critical habitats. Specifically, species experience a much greater change in habitat volume during Eastern Pacific (EP) El Niño events compared to Central Pacific (CP) El Niño or La Niña events, despite compensating effects of temperature and oxygen changes on metabolism during the former and the longer duration of the latter. Under future climate conditions, species with low hypoxia tolerance experience the greatest habitat variability, primarily driven by long-term warming-induced habitat loss. By the end of the twenty-first century, El Niño events no longer offset this decline, indicating a diminished capacity of these events to temporarily alleviate climate-related stress.

**Supplementary Information:**

The online version contains supplementary material available at 10.1038/s41598-025-06498-5.

## Introduction

The El Niño Southern Oscillation (ENSO), the most consequential fluctuation of the climate system, has been shown to impact ecosystems by inducing thermal stress on species and disrupting trophic interactions^1,2^. Although ENSO’s influence extends far beyond the tropical Pacific, its effects are most pronounced in this region due to the significant changes it induces in the oceanic and atmospheric circulation^3,4^. Impacts on fishery dynamics of commercially important species^5–12^, aquaculture and human societies^13,14^ have been widely accounted for.

The South Eastern Pacific (SEP) is a sensitive region to ENSO extending beyond the tropical Pacific encompassing the productive Humboldt Current System (HCS)^15–17^ and various chains of seamounts where pristine benthic ecosystems are found^18^. ENSO is characterized by Sea Surface Temperature (SST) anomalies either in the Eastern Pacific (EP) during EP El Niño or in the Central Pacific (CP) during CP El Niño or La Niña events^19^. During ENSO, temperature and dissolved oxygen in the SEP experience variations (Supplementary Figs. 1 and 2) through oceanic and atmospheric teleconnections acting on a combination of processes^20,21^. The SEP is also embedded into one of the largest Oxygen Minimum Zones (OMZ)^22^ which highly controls the species composition of the benthic and pelagic realms^23–25^. Rising temperatures during the warm phase of ENSO increase metabolic rates (MR) which scale with temperature^26^, reducing aerobic scope and therefore available oxygen to sustain ecological function such as growth and reproduction^27,28^. However, dissolved oxygen levels in the upper OMZ (oxycline depth) tend to increase during the warm ENSO phase^29–33^ which can compensate for the increase in temperature-driven oxygen demand defined by metabolic rates (MR). Therefore, changes in temperature and oxygen, or rather oxygen partial pressure (pO_2_)^34^ during ENSO may alter the balance between oxygen supply and demand with consequences on species distribution and abundance^35,36^.

The complexity of the impact of ENSO on the balance between O_2_ supply and demand further stems from the diversity of ENSO events—including cold and warm phases, strong and moderate intensities, Eastern Pacific (EP) and Central Pacific (CP) types, and variations in duration—collectively referred to as ENSO diversity^37^. This is further complicated by the fact that long-term warming affects both the balance between O_2_ supply and demand and the properties of ENSO itself. Notably, some types of ENSO events are projected to change under future climate conditions^38,39^. Two key questions thus motivate our analysis: (1) What is the degree of non-linearity in the SEP habitat response to warm and cold phases of ENSO—in terms of both magnitude and temporal evolution? In other words, are habitat gains or losses during El Niño events comparable to those during La Niña? (2) How might ENSO-driven changes in habitat be altered under future mean-state conditions and how do they compare to those due only to long-term changes? Specifically, could long-term warming counteract the beneficial effects of oxygenation typically observed during El Niño events?

**Fig. 1:**
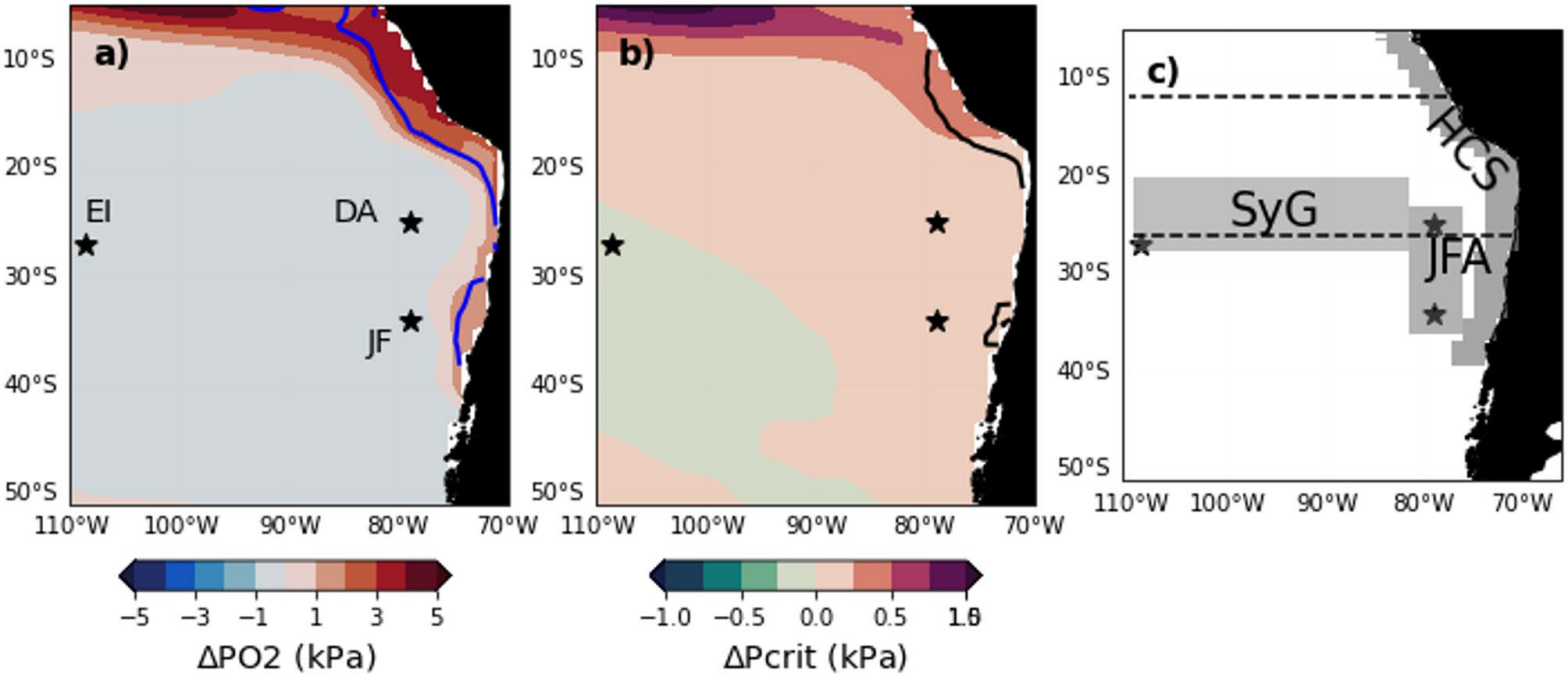
Average pO_2_ and P_crit_ anomalies associated with EP El Niño events at their peak in the historical scenario (1920–2005) at 100 m. Average pO_2_ (**a**) and P_crit_ (**b**) anomalies across 262 EP El Niño events associated with species with median levels of hypoxia tolerance (hereafter MHT species) corresponding to P_crit_ of ~ 4 kPa at T_ref_ (see Methods). The blue line in (**a**) is the 45 µmol oxygen isocontour at 100 m. The black line in (**b**) is where pO_2_ = P_crit_ at T_ref_ at 100 m. The horizontal dashed lines in (**c**) represent vertical sections at 12°S and 26°S. The stars represent the archipelago of Juan Fernandez (JF), Easter Island (EI) and Desventuradas (DA). The grey areas in (**c**) are subregions considered in the study: SyG for Salas y Gomez ridge, JFA for the Juan Fernandez and Desventuradas Island archipelagos and HCS for the Humboldt Current System region. Note the difference in scale in (**a**,**b**).

To address these questions, we examine the spatial and temporal patterns of changes in metabolically suitable marine habitats due to ENSO-induced potential mismatches between O_2_ supply and temperature dependant oxygen demand, across the contemporary (1950–2005) and future (2006–2100) climates as simulated by a state-of-the-art ESM model (Fig. 1a,b). We estimate changes in volume of suitable habitat (ΔVPO2_crit_, see Methods) occurring during extreme El Niño (EP EN) events and CP events including El Niño (CP EN) and la Niña (LN) events (see Methods) for species with varying degrees of hypoxia tolerance representative of the epipelagic and mesopelagic realms. Finally, we discuss changes in habitats due to EP El Niño in comparison to those driven by climate change (CC) in order to put in perspective the exposure due to ENSO in the future climate.

## Results

### Habitat change during ENSO in the present climate in the SEP

We first examine the effect of simulated 262 extreme El Niño events (EP EN), 540 CP El Niño (CP EN) and 940 La Niña events (See Methods and Supplementary Fig. 3) in the historical climate (1920–2005) in the domain 0-600 m, 5–50°S and 70–110°W. Habitat change is the result of ENSO-induced imbalances in oxygen supply and demand. While pO_2_ is used to approximate environmental oxygen supply (Fig. 1a, see Methods), P_crit_ is employed as the physiological oxygen threshold to approximate the temperature dependent oxygen demand (see Methods, Fig. 1b). Estimates of changes in habitat suitability between ENSO and neutral conditions are performed for the 3 species with low, median and high hypoxia tolerance (hereafter LHT, MHT and HHT species) (see Methods). We begin by presenting the spatial patterns of habitat changes along vertical sections at 12°S and 26°S. The 12°S transect was selected due to its location within a region strongly influenced by ENSO-driven hydrodynamic variability, while the 26°S transect intersects the Desventuradas and Salas y Gómez ridge seamount systems (see Fig. 1c). Next, we investigate the temporal dynamics using the integrative metric VPO2_crit_ that represents the volume of suitable habitat, defined as the water volume where pO_2_ > P_crit_ (VPO2_crit_). Changes in VPO2_crit_ (ΔVPO2_crit_) during ENSO events are calculated as the difference between VPO2_crit_ under neutral and ENSO conditions (see Methods, Supplementary Fig. 4).

**Fig. 2:**
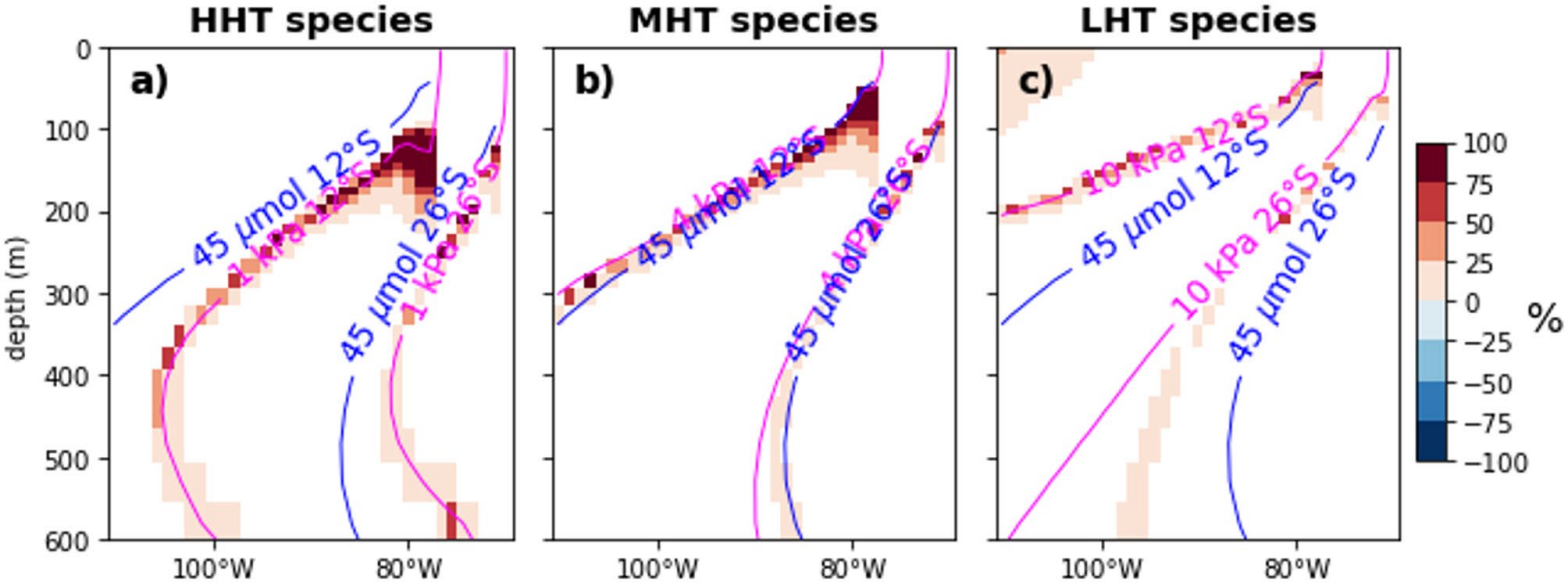
Probability density of changes in habitat suitability associated with the peak EP EN events in the present climate as a function of hypoxia tolerance levels at 12°S and 26°S. The probability density is presented as a percentage, the number of events when changes in habitat suitability occur divided by the total number of events. The red (blue) shading represents a gain (loss) of the cell, if the cell becomes metabolically suitable (unsuitable). Results at 12°S and 26°S are shown in the same panel. To locate the latitudes, the 45 µmol isocontours and the P_crit_ at T_ref_ are shown in blue and magenta lines, respectively.

Figure 2 (Supplementary Fig. 5) introduces the spatial pattern of changes in habitat suitability during EP EN (CP and LN events). It is presented as the probability density of changes i.e. as the percentage of events causing a change in habitat suitability across the total number of events. Consistently with known features in the ENSO teleconnection pattern in the SEP^40,41^, the probability changes in habitat principally occur in the oxycline with a greater centre of action in the upwelling centre. Figure 2 evidences a contrast between the coastal domain and the open ocean. In the case of EP EN, the probability density of changes in habitat suitability is more pronounced in the vicinity of the upper OMZ limit in the coastal upwelling area (> 75% of events) where suitable habitat expands vertically by a few dozen meters near the coast (Supplementary Fig. 6 and 7). ΔVPO2_crit_ essentially occurs north of 20°S and in the coastal domain from the coast to ~ 90°W (Supplementary Fig. 6). It is also stronger between the surface and 400 m with peak ΔVPO2_crit_ between 100 and 200 m (Supplementary Fig. 6). The limit of viable habitat may vary by at least 10 m during EP EN (Supplementary Fig. 7). CP EN and LN events exhibit comparable spatial patterns (Supplementary Figs. 5, 6 and 7).

**Fig. 3:**
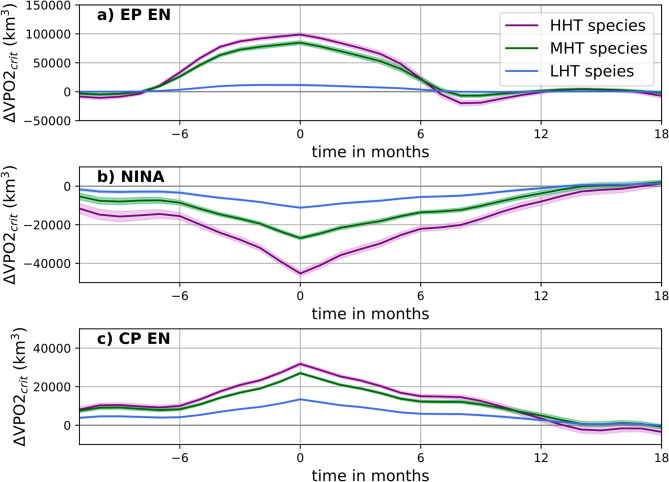
Composite evolution of the net change in habitat (ΔVPO2_crit_) in the SEP during ENSO in the present climate. Composite evolution of ΔVPO2_crit_ (km^3^) (EP EN in (**a**), CP EN in (**b**) and La Niña in (**c**) for HHT, MHT and LHT species, in the historical scenario (1920–2005). The envelope (shading) represents ± the standard deviation amongst 10,000 composites generated randomly using a bootstrapping method (see Methods). Time in months to the peak (0) of the event.

The changes observed along these two sections mirror those found across the OMZ’s meridional gradient, with differences in amplitude and vertical extent driven by variations in oxycline depth and sharpness, as well as ENSO-induced extratropical Rossby waves dynamics^41^. The cumulative impact on habitat suitability is effectively captured by ΔVPO2_crit_. We now present the results using the integrative metric ΔVPO2_crit_ in order to compare the temporal evolution of changes in habitat across events and climates (Fig. 3).Fig. 3 first indicates that EN events produce a gain of habitat whereas LN produces a loss of habitat. The sign of the change is explained by the more sensitive response of ΔVPO2_crit_ to ΔpO₂, which outweighs the non-linear effects of variations in temperature. Along the Z_crit_, the depth at which pO₂ equals P_crit_ (marking the limit of habitat suitability), ΔpO₂ accounts for ≥ 50% of the observed ΔVPO2_crit_ in the upper oxygen minimum zone (OMZ) (Supplementary Fig. 8). During EN events, the pronounced warming of the thermocline increases metabolic oxygen demand, but this effect is compensated for by the rise in oxygen supply (positive ΔpO₂). Conversely, during LN events, subsurface cooling reduces oxygen demand, but this is offset by a decrease in pO₂, resulting in a negative net balance between oxygen supply and demand. These outcomes are sensitive to physiological traits.

Changes in oxygen demand have been assessed using a median E_0_ value of 0.34 eV, where E_0_ represents the temperature sensitivity of oxygen demand and physiological oxygen supply^42^. However, this parameter can vary between − 0.1 eV to 0.9 eV across a diversity of species^42^. To account for this variability, we explored the full range of E_0_ values to assess its influence on the temperature contribution to VPO2_crit_ (Supplementary Fig. 9). Supplementary Fig. 9 shows that the sign of ΔVPO2_crit_ is only partially controlled by E_0_. While the prevalence of variations in oxygen (i.e. O₂ supply) over temperature (i.e. O₂ demand) remains for HHT and MHT species regardless of E_0_ (Supplementary Fig. 9ab), temperature starts to be the driver of ΔVPO2_crit_ for LHT species from an E_0_ value of 0.4 eV (Supplementary Fig. 9c).

**Fig. 4:**
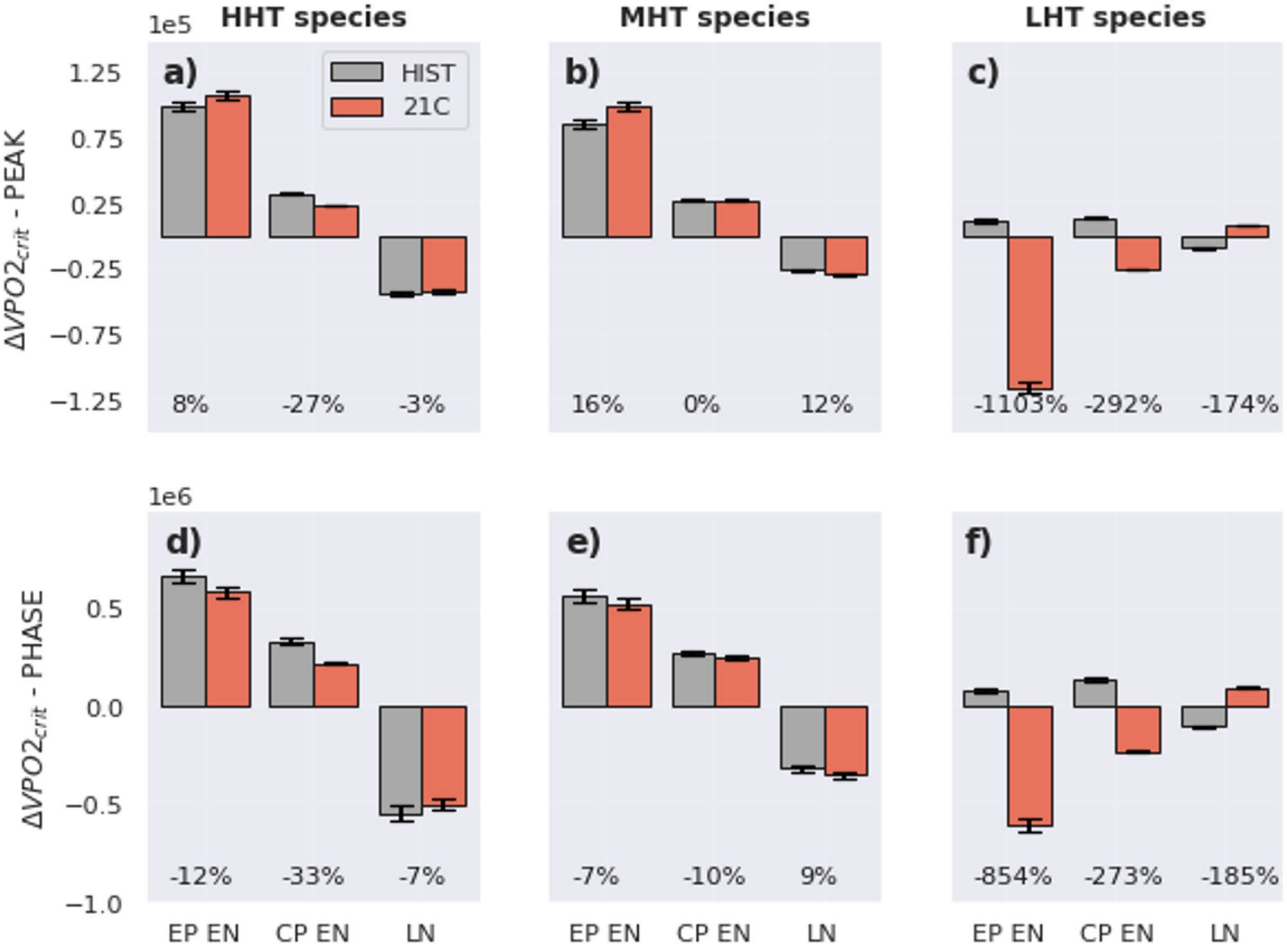
Composites of ∆VPO2_crit_ during EP and CP events in the (HIST) historical and (21C) future climates. (top panels) ∆VPO2_crit_ at the peak of ENSO and (bottom panels) ∆VPO2_crit_ integrated over the ENSO phase, i.e. during the periods when ENSO indices are above a certain threshold (see Methods). In the historical (future) climates, and across the 34 members, CESM-LE simulates: 262(305) EP EN, 540(675) CP EN and 940(1116) LN. The % indicates the variation of ∆VPO2_crit_ in the future climate compared to the historical climate. The error bars are the standard deviation amongst 10,000 composites generated randomly using a bootstrapping method (see Methods).

Figures 3 and 4a–c (grey bars) also reveal an amplitude asymmetry in ΔVPO2_crit_ during the peak of ENSO events regardless of the species. EP EN has a greater impact on the volume of suitable habitat compared to CP events and favours species with higher degrees of hypoxia tolerance (+ 300% for HHT at the peak). The change in volume due to EP EN is about twice the change in volume due to CP events in the historical climate for the three (HHT, MHT, LHT) species combined: EP EN produces a gain of 1.8e5 km3 while LN(CP EN) produces a loss(gain) of 1e5 km3. These differences in amplitude are consistent with the intrinsic asymmetry of ENSO, whereby EP EN end to exhibit much stronger amplitudes than both CP EN and LN events^19^.

Beside the amplitude asymmetry of ΔVPO2_crit_, Fig. 3 also reveals a temporal asymmetry between EP EN and LN events, with habitat loss during LN lasting longer than habitat gain during EP EN. This arises from the temporal asymmetry of ENSO^43^, which manifests as La Niña events having a tendency to linger over two to three years, particularly after strong El Niño events^44^.

The effect of this temporal asymmetry is further diagnosed in Fig. 4d–f (grey bars ΔVPO2_crit-phase_ in Fig. 4d–f) where ΔVPO2_crit_ is cumulated over the developing phase of EP and CP events (Supplementary Fig. 5). When considering the duration of events, the impact of EP EN remains greater than that of CP EN or LN, despite their greater duration for MHT and LHT species. However, the impact of LN in terms of volume either approximately balances or surpasses that of EP EN in the historical climate for HHT species (grey bars Fig. 3d–f). This suggests that the temporal asymmetry of ENSO—specifically, the tendency for La Niña events to persist longer than El Niño—does not always compensate for their low magnitude. For CP El Niño events, the cumulative volume change is approximately half of that observed during EP EN events for high- and medium-tolerance species. However, for low-tolerance species, the impact of CP events exceeds that of EP events due to longer lasting CP events.

Differences in the frequency of occurrence between ENSO event types may also influence habitat dynamics in distinct ways—an effect that can itself be modified by long-term warming. To evaluate this, we can multiply the cumulative ΔVPO2_crit_ during the development phase of each ENSO type (ΔVPO2_crit-phase_, grey bars Fig. 4d–f) by the decadal frequency of occurrence of each event (Table 1). In the present climate, LN occur about three times more often than EP EN. As a result the total change in volume associated with LN is between 1.5 times (MHT species) and 3.9 times (LHT species) greater than that of EP EN despite EP EN events producing larger ΔVPO2_crit-phase_ values. A similar pattern is observed for CP events, for which cumulative ΔVPO2_crit-phase_ may roughly match (for HHT and MHT species) or exceed (> 2 times for LHT species) that of EP EN.

**Table 1:** Average frequency of occurrence per decade of ENSO events.

	EP EN	CP EN	LN
HIST (1920–2005)	0.91	1.86	3.24
RCP 8.5 (2006–2100)	0.94 (+ 4.8%)	2.13 (+ 14%)	3.5 (+ 8%)

The frequency of occurrence is the total number of events divided by the number of decades in each climate period. The number in parenthesis indicates the change in frequency between the future and historical climates, which is significant at the 95% level based on a bootstrapping method.

### Habitat change during ENSO in the future climate in the SEP

We now examine the evolution of ΔVPO2_crit_ under future climate conditions (2006–2100), which are shaped by changes in both ENSO characteristics (intensity, duration, and frequency) and the mean climate state. Since P_crit_ responds non-linearly to temperature, shifts in mean conditions also influence its sensitivity to ENSO variability. This section begins by documenting how changes in ENSO properties (amplitude, temporal evolution and frequency changes) affect habitat in a warmer climate. We then focus on EP El Niño events to explore the non-stationarity of these ENSO-driven habitat variability by comparing two distinct periods of the twenty-first century. This approach allows us to disentangle and contrast the impacts of long-term warming and ENSO variability on habitat change. To enhance the real-world applicability of our findings, our analysis also targets three regions of both ecological and economic importance.

Figure 4a–c (red bars) indicates that peak ΔVPO2_crit_ varies significantly in the future climate and exhibits the greatest variations for LHT species. ΔVPO2_crit_ switches sign from a gain to a loss of habitat equivalent to − 1000% (Fig. 4c). ΔVPO2_crit_ also switches sign during CP EN and La Niña events with ΔVPO2_crit_ varying by + 200%. Variations in ΔVPO2_crit_ during the peak of EP EN are also significant for HHT species however to a lower extent compared to LHT species. MHT species experience the lowest variations in ΔVPO2_crit_ across climates.

When considering the integral of ΔVPO2_crit_ over the ENSO developing phase (i.e. ΔVPO2_crit-phase_ in Fig. 4d–f), changes in habitat between climates show more contrasted results. In the future climate, LHT species (Fig. 4f) experience a significant decrease in ΔVPO2_crit-phase_ during the phase of EP EN (-800%) and HHT species during LN (-200%). On the other hand, ΔVPO2_crit-phase_ decreases during EP EN for HHT species suggesting the effect of shorter but more intense EP EN. Interestingly, ΔVPO2_crit-phase_ during ENSO does not vary significantly for MHT species in the future climate (Fig. 4e) indicating that variations in the balance in oxygen supply and demand across climates do not vary significantly at the limit of their habitat. This result suggests that species with “extreme” hypoxia tolerance (HHT and MHT) will be more sensitive to ENSO in the future climate.

Given that ENSO events are projected to become more frequent in a warmer climate—a trend captured by CESM-LE—changes in ΔVPO2_crit-phase_ between present and future climates can be used to estimate the cumulative habitat impact of this increasing frequency. This is done by multiplying the per-event ΔVPO2_crit-phase_ by the projected change in the frequency of occurrence of each ENSO type. In agreement with previous studies^38,39,44–46^, CESM-LE simulates an overall increase of at least ~ 5% in the frequency of all ENSO types (see Table 1 for details). Our results thus suggest that, for this model, cumulative changes in ΔVPO2_crit-phase_ will be most exacerbated by the frequency changes in events for CP events (+ 14%), followed by LN events (+ 8%).

We now compare habitat changes induced by ENSO events with those driven by long-term trends, taking into account the non-stationarity of both ENSO characteristics and the mean climate state. Due to the non-linear dependence of P_crit_ on baseline environmental conditions, the modulation of ENSO-related habitat changes is inherently complex and not readily predictable. To capture this evolving behaviour, we divide the twenty-first century into two sub-periods: 2006–2050 (‘BEG_21C’) and 2050–2100 (‘END_21C’). These timeframes reflect the progression of climate change and can be interpreted as analogues of different warming levels (see Supplementary Fig. 4 and Methods for details). Since EP EN are far more intense than CP or LN events at their peak, and that the ecological impact of the longer duration or higher frequency of CP EN and LN events cannot be assessed here, we now chose to focus exclusively on EP EN. Lastly, we distinguish three economically and ecologically relevant subregions (Fig. 1c): the HCS coastal and upwelling regions off Peru and Chile, the Juan Fernandez and Desventuradas archipelagos (JFA) and the seamount system of Salas y Gomez (SyG) which are characterized by contrasted climate changes and ENSO teleconnection patterns. In particular long-term reoxygenation is confined along the coast of Peru and Chile in the upper bound of the OMZ^29^, whereas long-term warming takes place at basin-scale but with a stronger rate in the tropical region of the SEP and extends vertically into the sub thermocline^40^. With regard to ENSO teleconnections, it is primarily the coastal zone that experiences significant warming during strong El Niño events^4^. This warming is driven by planetary waves of equatorial origin that propagate poleward along the coast while also extending westward and downward into the ocean interior^41^. This implies different sensitivities of ΔVPO2_crit_ across regions, depth and subsequently species.

**Fig. 5:**
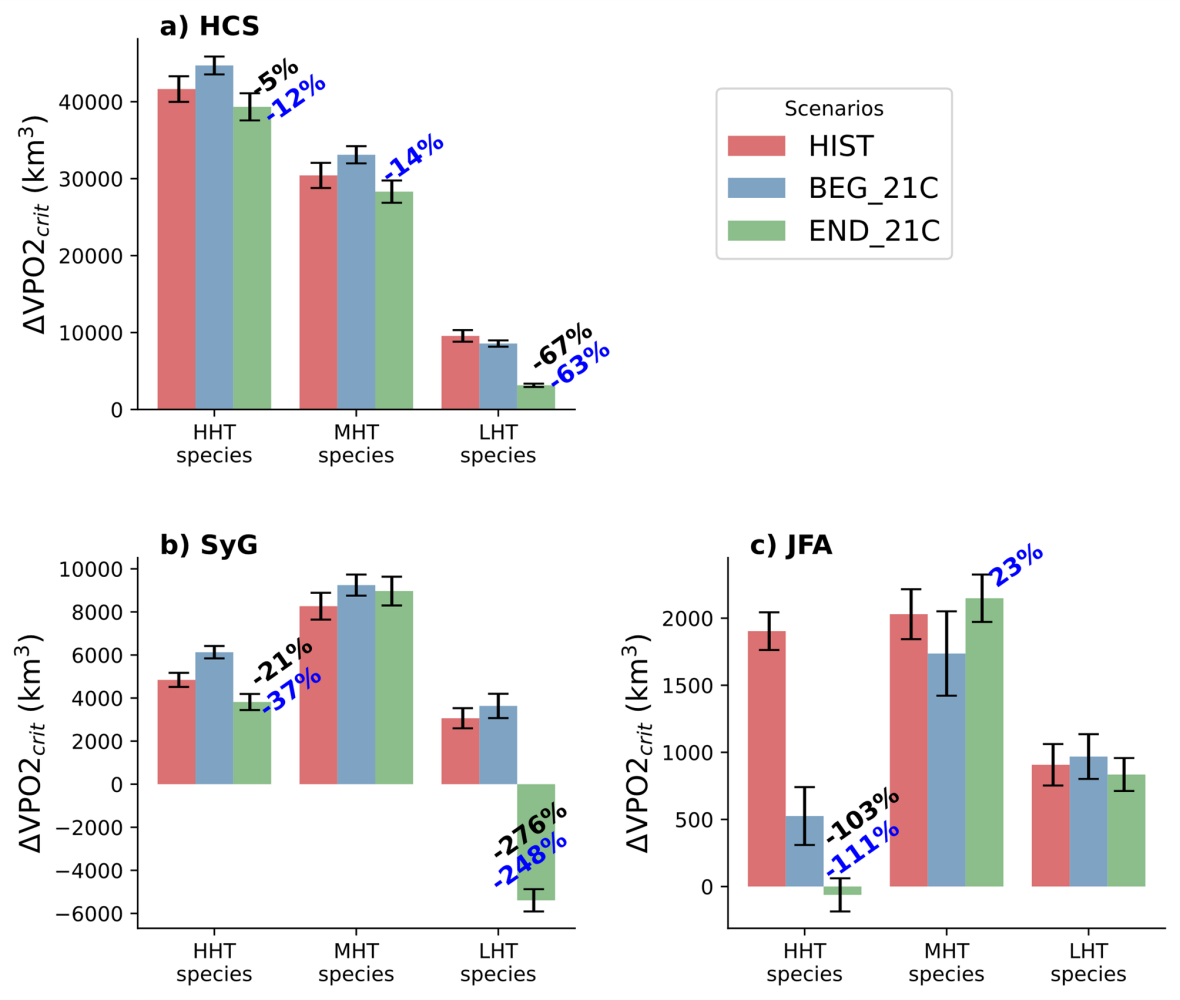
Mean change in suitable habitat volume (ΔVPO2_crit_) during the peak of El Niño across climates and regions. The ‘HIST’ period covers the years 1950–2005, the BEG_21C period the years 2006–2050 and the END_21C period the years 2050–2100. (**a**) the Humboldt Current System (HCS), (**b**) Salas y Gomez ridge (SyG) and (**c**) the Juan Fernandez archipelago (JFA) (see Fig. 1c for domains). The vertical black bars represent the error estimated as the standard deviation of resampled ΔVPO2_crit_ EN composites using a bootstrapping method (see Methods). The % indicates the change in ΔVPO2_crit_ (%) between periods where it is significant, in black: ‘END_21C’ period relative to the historical period and in blue: ‘END_21C’ relative to ‘BEG_21C’.

Figure 5 indicates that the ΔVPO2_crit_ exhibits the greatest variations in comparison to the historical climate during the END_21C period. This is particularly the case for the seamount systems where LHT species in the SyG region and HHT species in the JFA regions have the most significant drop in ΔVPO2_crit_ : ~ -250% and ~ -100%, respectively. Interestingly, ΔVPO2_crit_ increases in the HCS and SyG in the BEG_21C period, but drops back to at least historical levels in the END_21C period, which illustrates the non-linear response of ENSO-induced habitat changes to long-term warming. These results can be contrasted with estimates of the changes in ΔVPO2_crit_ due to ENSO between future and present climates when the effect on P_crit_ due to long-term warming and the effect of deoxygenation are removed (Supplementary Fig. 10). Long-term trends in oxygen and temperature induce variations in habitat suitability in neutral conditions which have a subsequent impact on the limit of habitat suitability described by Z_crit_. Variations in Z_crit_ (Supplementary Fig. 11) due to long-term warming and deoxygenation thus modify the sensitivity of species to ENSO-induced anomalies. In particular HHT and LHT species in all regions are more affected by long-term trends in temperature (Supplementary Fig. 12). However, MHT species remain in a zone where the influence of the long-term trends is limited (Supplementary Fig. 12).

**Fig. 6:**
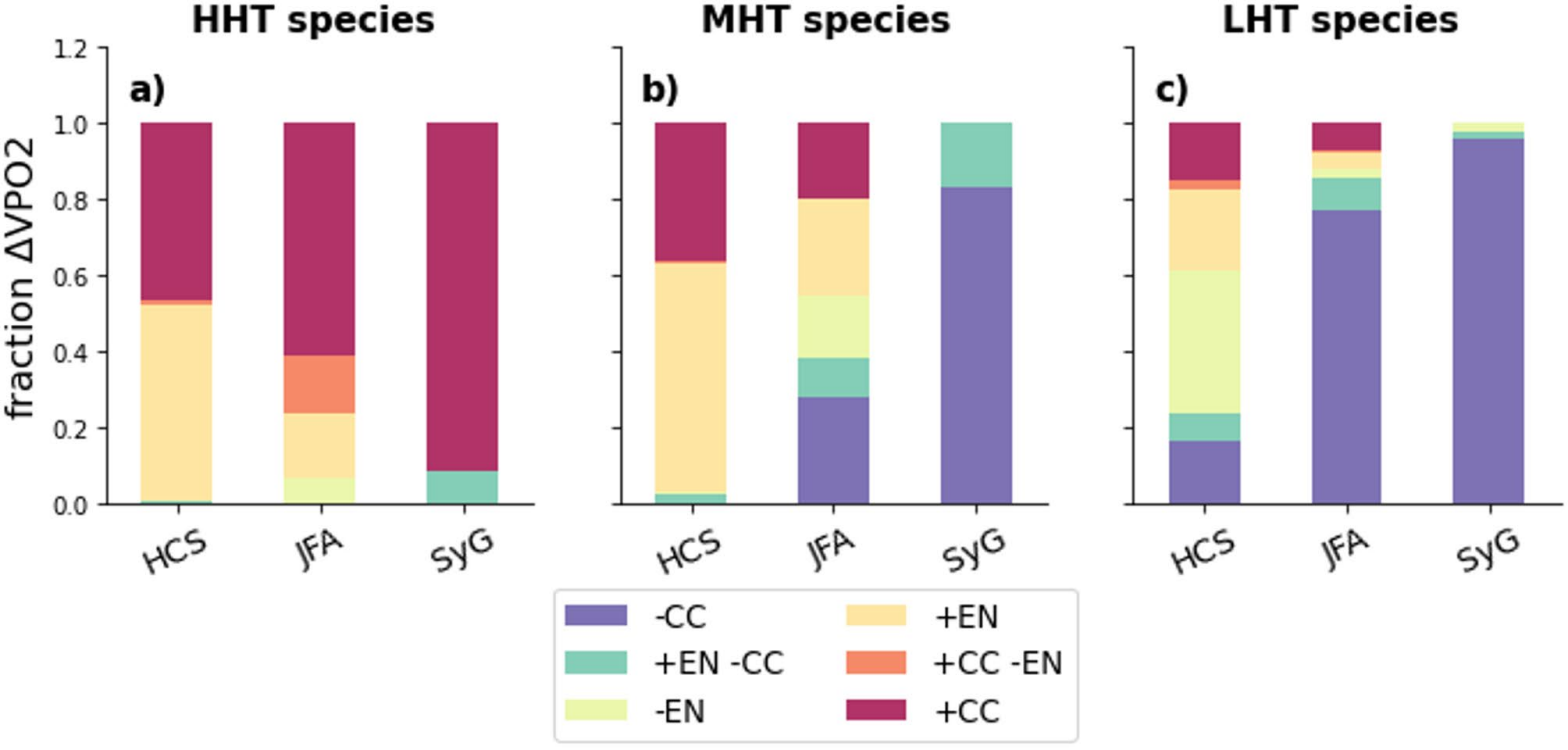
ΔVPO2_crit_ due to EN or CC in the END_21C period across regions. Fractions of ΔVPO2_crit_ were calculated within several key regions of the SEP (see Fig. 1c for the definition of the region). We identify if CC or EN produces a gain or loss of suitable habitat (VPO2_crit_) in each sub-region. It leads to 6 different combinations of gain/loss due to EN and/or CC. A “ + ”(“ − ”) sign means that EN or CC cause a gain (loss) of VPO2_crit_. For instance, the combination “ + CC -EN” means that habitat is gained due to climate change but is lost during EN. The fraction is estimated by normalizing (i.e. dividing) ΔVPO2_crit_ by the sum of ΔVPO2_crit_ for the 6 combinations. Hence fractions vary between 0 and 1 and allow to compare the subregions.

Finally we evaluate the relative importance of habitat gain (or loss) due to EN and CC. In particular, from the above, we question if the effect of EP EN on HHT species (mostly gain) will prevail over the CC effect by the end of the twenty-first century and if additive or compensating effects can take place. In order to assess this, we present in Fig. 6 the fraction f (and sign) of ΔVPO2_crit_ due to CC and/or EN in each subregion (see associated spatial pattern at different depths in Supplementary Fig. 13). Our results indicate that across most regions and species the magnitude of the change in habitat due to CC will surpass that due to EN. In fact, CC mostly drives a loss of suitable habitat that EN only partially compensates (Fig. 6a). The impact of EN is however greater than CC in the northern HCS for high- and median hypoxia tolerant species (Fig. 6a,b, Supplementary Fig. 13de). Furthermore, EN and CC have additive effects on ΔVPO2_crit_ in the HCS for HHT species (Fig. 6a,b). Such an additive effect decreases with lower hypoxia tolerance levels (Fig. 6c). Lastly, except for the case of HHT species, the impact of EN is negligible in the seamount regions. In conclusion, CC or EN are expected to improve the habitat suitability of the most hypoxia tolerant species whereas MHT and LHT species will be negatively impacted in the seamount systems in particular.

## Discussion and concluding remarks

ENSO profoundly impacts the SEP environment by modulating oxygen and temperature levels, but how it impacts marine aerobic habitats around key areas of the SEP has remained unclear limiting our capacity to design adaptation strategies in a region that hosts one of the most unique and biodiverse seascapes on Earth with a high rate of endemism and over 80 threatened or endangered species^18^. Here we addressed this issue based on P_crit_, which accounts for minimal metabolic demand. As ENSO produces mismatches between oxygen supply and oxygen demand, we captured the temporal pattern of these imbalances looking at changes in volume of critical habitat (VPO2_crit_). Our results first showed a marked amplitude asymmetry between EP EN and CP EN or La Niña where EP EN impacts species twice as much as La Niña except for shallow species for which changes in VPO2_crit_ have similar amplitude (Fig. 3). The temporal asymmetry of ENSO events also reflects on ΔVPO2_crit_ where the cumulative ΔVPO2_crit_ over the developing phase of EP EN and LN exhibit comparable magnitude for HHT species despite their differences in peak amplitude in terms of ΔVPO2_crit_ (Fig. 4d–f). Since the frequency of occurrence of LN is larger than that of EP EN (Table 1), this means that the cumulative change in aerobic habitat during LN (overall loss) over a fixed period of time is superior to that of EP EN (overall gain). This implies that the temporary refuge provided during EP EN could be a buffer against the habitat loss generated during LN. Another important finding is the role of the long-term trends in modulating the impact of ENSO. Our results indicate that across most regions and species the magnitude of the change in habitat due to CC will surpass that due to EN (Fig. 6). Besides, the impact of ENSO or CC in the future climate was found more pronounced for HHT and LHT species (Figs. 5 and 6). This has potentially important implications for ecosystem functioning causing a decoupling between organisms evolving in the epi- and mesopelagic layers. It could also further affect fishing or aquaculture as epipelagic organisms which make up the majority of the fishing resources will likely be driven out of their present habitat. Hence, in the future climate, either due to EN or CC, only a small fraction of the SEP (i.e. the upper OMZ) would constitute a limited climate refuge^47^.

While our study is the first to rely on physiological metrics to assess habitat changes during ENSO in the SEP, it does have some methodological limitations that need to be discussed. First, we have used SMR as a threshold for aerobic metabolism. However, this does not account for the energy needed for ecological functions such as growth and reproduction which enable it to support populations. For the SEP, the data to account for ecological activities (i.e. maximum metabolic rates) is lacking thus restricting the ability of our approach to estimate aerobic habitat changes considering ecological functions.

Second, our study focuses on single species whereas individual responses may significantly reverberate at the population and community levels especially in a context of a decoupling and could be best captured using a holistic approach considering multiple timescales of variability in the environmental forcing^48^ and/or ecosystem models^49,50^. In particular, the contrasting impacts of EN on the epipelagic and mesopelagic layers could pose significant challenges, particularly given the importance of vertical coupling in trophic interactions. Our results also suppose that species will not adapt to future temperature and oxygen levels or fluctuations. However, there is evidence that some species could reduce their metabolic demand under warming using phenotypic plasticity^51–53^. That said, capacity for plasticity appears limited^54^, only partially beneficial and also highly variable among species^55^. In fact, very little is known about the adaptive potential of species^56^.

Despite these limitations, our study highlights that EP EN enhances aerobic habitat in the SEP. Since EP EN often precede multi-year LN episodes, we hypothesize that they may serve as a buffering mechanism, mitigating the negative impacts of LN on aerobic habitats by enabling a prior expansion of favourable conditions that support population growth and/or health before the subsequent habitat compression. Determining whether such a mechanism can be substantiated through ecological modelling warrants further investigation, though this lies beyond the scope of the present study. Our study finally revealed that the positive effect of EP EN diminishes significantly by the second half of the twenty-first century due to long-term climate trends. The observed interaction between extreme events and persistent trends in shaping aerobic habitat expands the current framework for understanding compounded impacts on ecosystems in the SEP.

## Data and methods

### Model data

We use the data produced by the NCAR Community Earth System Model Large Ensemble Project (CESM-LE)^57^ from the historical and RCP 8.5 scenarios covering the years 1920–2005 and 2006–2100, respectively. We use temperature, oxygen and salinity data from 34 members of the model with a 1° × 1° resolution over the 70–110°W and 5–50°S domain. pO_2_ (in mbar) is calculated using temperature and salinity with the formula from^58^. This model resource has been widely used for climate studies due to the skill of the model in accounting for many aspects of the climate variability^59,60^, including ENSO^61,62^ as well as biogeochemical properties^63^. Additionally, the availability of numerous realizations of the same climate scenario enables the generation of robust statistical analyses.

### Selection of ENSO events

ENSO can be divided into two main regimes^37^: one regime associated with extreme warm events for which SST anomalies peak in the far eastern Pacific extending along the coast of Peru and northern Chile, and another regime associated with peak central Pacific SST anomalies^19^. We focus here on extreme El Niño events that are of Eastern Pacific (EP) type^19^ because they exhibit a marked oceanic teleconnection along the coasts of Peru and Chile^4,61^. La Niña events tend to be centred in the central Pacific and are generally weaker in magnitude compared to strong El Niño events, which typically peak in the eastern Pacific—this contrast contributes to the positive asymmetry of ENSO^64^.

We use the Niño-3.4 SST index to detect ENSO events using the method from^44^. El Niño and La Niña events are events for which SST anomalies over October November December January February (ONDJF) in the Niño-3.4 region (5°S-5°N, 120°W-170°W) are above 1.5 s.d., and below − 0.5 s.d. of the Niño 3.4 SST values, respectively. Using this method we identified 264(305) EP El Niño, 540(682) CP El Niño (CP EN) , 939(1119) and La Niña in the historical(RCP 8.5) scenario, respectively.

### pO_2_ and temperature patterns associated with ENSO events

To construct oxygen and temperature anomaly patterns during El Niño and La Niña events, we use the E and C indices which are independent by construction (Eq. 1). This allows us to calculate the spatial and temporal patterns of any variable due to El Niño and La Niña independently (Eq. 2). The E and C indices^19^ characterize time series of SST anomalies associated with ENSO events, where E and C (Eq. 1) are defined as :

1 $${\text{E}}\left( {\text{t}} \right) = \frac{{{\text{PC}}1 + {\text{PC}}2}}{\sqrt 2 }\;{\text{and}}\;{\text{C}}\left( {\text{t}} \right) = \frac{{{\text{PC}}1 - {\text{PC}}2}}{\sqrt 2 }$$

where PC1 and PC2 are the principal components of the first two EOF modes of SST anomalies in the tropical Pacific (120°E-290°E; 10°N-10°S). E and C (dimensionless) are the times series of SST anomalies patterns characterising the two modes of SST variability in the Eastern Pacific (EP) and Central Pacific (CP), respectively.

We use the E- and C-indices corresponding values for the events detected with the Niño-3.4 index to produce the composite time series associated with El Niño and La Niña events (Supplementary Fig. 3).

pO_2_ and temperature anomalies were calculated using the E and C time series for each event. pO_2_ and temperature anomalies associated with ENSO events are the projection of the coefficients α and β of the bilinear regression of pO_2_ or temperature onto the E and C times series (Eq. 2) so that:

2 $${\text{PO}}_{2} = \upalpha_{{{\text{PO}}2}} {\text{E}} + \upbeta_{\text{PO2}} {\text{C}}$$

### Oxygen demand and $\text{P}_{{\rm crit}}$ formulation

Oxygen demand is defined by metabolic rates which represent the levels of oxygen needed to support a variety of physiological processes and ecological functions. Metabolic rates vary between Standard Metabolic Rates (SMR) and Maximum Metabolic Rates (MMR). P_crit_ describes the minimum level of oxygen necessary to sustain SMR^65^. Consequently, variations in environmental oxygen or temperature-driven changes in MR may modulate available aerobic habitat. Conceptualised as the ratio of oxygen supply to oxygen demand (Eq. 3), the Metabolic Index (Φ) framework^27,42^ captures such capacity of the environment to sustain aerobic metabolism, providing the environment is oxygen limited^66^.

3 $$\Phi = \frac{{{\text{PO}}_{2} \;{\text{supply}}}}{{{\text{PO}}_{2 } \;{\text{demand}}}} = \frac{{\upalpha_{{\text{S}}} }}{{\upalpha_{{\text{D}}} }} \times {\text{PO}}_{2} \times {\text{B}}^{{\text{n}}} \times {\text{e}}^{{\frac{{{\text{E}}_{0} }}{{{\text{k}}_{{\text{B}}} }}\left( {\frac{1}{{\text{T}}} - \frac{1}{{{\text{T}}_{{{\text{ref}}}} }}} \right)}}$$

where k_B_ is the Boltzman constant (j/K). B is the body mass and n the allometric exponent, which can be set equal to 1 (Penn et al., 2018). $\frac{{\alpha }_{S}}{{\alpha }_{D}}$ is the ratio of the oxygen supply capacity (µmol O_2_g^-3/4^ h^-1^ atm^-1^) and oxygen demand (µmol O_2_g^-3/4^ h^-1^), i.e. resting metabolic rates. The ratio $\frac{{\alpha }_{S}}{{\alpha }_{D}}$ is equal to A_0_ (in atm^-1^), the hypoxia tolerance threshold, and is the inverse of P_crit_ at a reference temperature T_ref_ = 15 °C when Φ = 1^42^; Eq. 3). E_0_ (eV) is the sensitivity to temperature of ${\alpha }_{S}$ and ${\alpha }_{D}$ and therefore of A_0_.

The metabolic index (MI) is a sensitive framework to use for several reasons. First and foremost, it does not account for oxygen limitation of MMR which can potentially increase indefinitely with pO₂^66^. Furthermore, through the ecological threshold Φ_crit_ which reflects the minimum energy required for ecological activities^27^, the MI framework has demonstrated some skills in establishing a correlation between biogeography and metabolic requirements^42,67^ although not always^68^. Moreover, the correlation may be an artefact of the pO₂-temperature phase space^66^. Finally, the ecological threshold Φ_crit_ used in biogeographic studies is not known for SEP species, thus introducing an additional degree uncertainty when inferring habitat changes based on this threshold (see Supplementary Figure S9 in ref.^47)^.

For these reasons, we chose to use P_crit_ to assess changes in oxygen supply and demand. P_crit_ can be derived from the formulation of the metabolic index Φ^42^; (Eq. 3) to account for the effect of temperature T. When oxygen supply equals oxygen demand (Φ = 1), we obtain the formulation for P_crit_ (Eq. 4):

4 $${P}_{crit}=\frac{1}{{A}_{0}}\times {e}^{\frac{-{E}_{0}}{{k}_{B}}\left(\frac{1}{T}-\frac{1}{{T}_{ref}}\right)}$$

In our study, oxygen supply refers to the environmental oxygen supply (pO₂) and oxygen demand is represented by P_crit_. The thermal sensitivity of MR and the physiological oxygen supply are encapsulated in the parameter E_0_ .

### Physiological traits

The traits A_0_ and E_0_ are available for 71 species (see database in ref.^42^). We do not select species reported in the SEP as they represent a very limited sample (8 species using www.obis.org, www.aquamaps.org), not representative of the diversity of species, and whose traits were measured on species collected in other ocean basins. Instead, we use a minimum (10th percentile), median and maximum (90th percentile) values (10, 23, 67 atm^-1^) of the 71 species from which we derive P_crit_.

This way we obtain 3 thresholds of P_crit_ equal to 1, 4 and 10 kPa at T = T_ref_ (Eq. 4), representing species of high, median and low hypoxia tolerance (hereafter HHT, MHT and LHT species). For E_0_ we take the median value of 0.34 eV (median of E_0_ of the 71 species). To examine the impact of various E_0_ values, we take the full range of the E_0_ distribution (-0.1 to 0.9 eV)^42^.

### Definition of critical habitat volume (VPO2_crit_) and its change (ΔVPO2_crit_)

Because the distribution of species with the selected P_crit_ values cannot be known, we define as aerobic habitat the volume for which pO_2_ > P_crit_ that is $VPO{2}_{crit}=\sum \left(PO{2}_{i,j}-Pcri{t}_{i,j}\left(T\right)\right)>0$. The changes in habitat between two states (S1 and S2) is then defined by the change in volume:

5 $$\Delta VPO2_{crit} = VPO2_{crit - S2} {-}VPO2_{crit - S1}$$

In Fig. 2, a cell (i,j) is considered metabolically suitable when pO_2_(i,j) > P_crit_(i,j). Change in habitat suitability occurs when a cell becomes or is no longer suitable.

### pO_2_ and temperature climatological means

Climatological mean of pO_2_ and Temperature (hereafter pO_2neutral_ and T_neutral_) corresponds to data averaged over ONDJF and further averaged over the duration of the subperiods. For instance, pO_2clim_ over 1920–2005 is $$PO{2}_{neutral}=\frac{1}{N}$$$${\sum }_{i=1920}^{N=2005}\underset{\_}{PO{2}_{ONDJF}}$$, and equivalently for temperature.

### pO_2_, temperature and $\text{P}_{{\rm crit}}$ during ENSO

pO_2_ and temperature during ENSO correspond to the sum of the anomalies due to ENSO and the climatological mean (pO_2neutral_ and T_neutral_) of the reference periods. So that pO_2_ and temperature during ENSO are $P{O2}_{ENSO}=PO{2}_{neutral}+{\Delta }_{ENSO}$ and ${T}_{ENSO}={T}_{neutral}+{\Delta }_{ENSO}$.

### Subperiods and levels of surface warming

We study three periods covering the years 1920–2005, 2006–2050 and 2050–2100 named ‘HIST’, ‘BEG_21C’ and ‘END_21C’, respectively. We calculate the warming associated with the ‘BEG_21C’ and ‘END_21C’ subperiod relative to ‘HIST’ to represent levels of surface warming in the SEP (Supplementary Fig. 4). Average surface warming of the SEP associated with the ‘BEG_21C’ and ‘END_21C’ periods are of + 0.6 and + 1.7 °C, respectively. Since ‘low’ warming levels can be reasonably derived from high emission scenarios^69^, the future evolution of ENSO events at different time periods can be used to infer changes due to warming levels^70^.

### Statistical significance of tests of composites

We use a bootstrap method to estimate if the El Niño and La Niña composites are statistically significant. It consists in randomly selecting 10,000 subsets of events. The subsets are composed of 50% of the total number of events. Events are allowed to be re-selected. Then the standard deviation is calculated across these 10,000 realisations. For instance, for the ΔVPO2_crit_ of EN in Fig. 2, we generate 10,000 subsamples of 131 events (half of the 262 events) and calculate the standard deviations amongst the 10,000 derived composites.

## Electronic supplementary material

Below is the link to the electronic supplementary material.

Supplementary Material 1

## Data Availability

Oxygen, temperature and salinity data are available at https://www.cesm.ucar.edu/community-projects/lens2/data-sets. The data and scripts used to compute the figures are available at https://github.com/aparouffe/ENSO.
