# Supplementary material for: ENSO modulates aerobic habitat across varying hypoxia tolerance levels in the Southeast Pacific throughout the twenty-first century
Source: Sci Rep. 2025 Jul 1;15:20670. doi: 10.1038/s41598-025-06498-5 (PMC12219086; doi:10.1038/s41598-025-06498-5)
Supplement: Supplementary file 1 — Supplementary Material 1 [file 41598_2025_6498_MOESM1_ESM.docx]

**ENSO modulates aerobic habitat across varying hypoxia tolerance levels in the Southeast Pacific throughout the 21st century**

Parouffe, A.1*, Dewitte, B.2,3,4, Paulmier, A.1 and, Garçon, V.5

1 Laboratoire d’Etudes en Géophysique et Océanographie Spatiales, Université de Toulouse, LEGOS (CNES/CNRS/IRD/UPS), Toulouse, France

2 Centro de Estudios Avanzados en Zonas Aridas (CEAZA), Coquimbo, Chile

3 CECI, Université de Toulouse, CERFACS/CNRS, Toulouse, France

4 Departamento de Biología, Facultad de Ciencias del Mar, Universidad Católica del Norte, Coquimbo, Chile

5 CNRS/IPGP, Institut de Physique du Globe de Paris, Paris, France

**Supplementary material**

**Supplementary Figures**


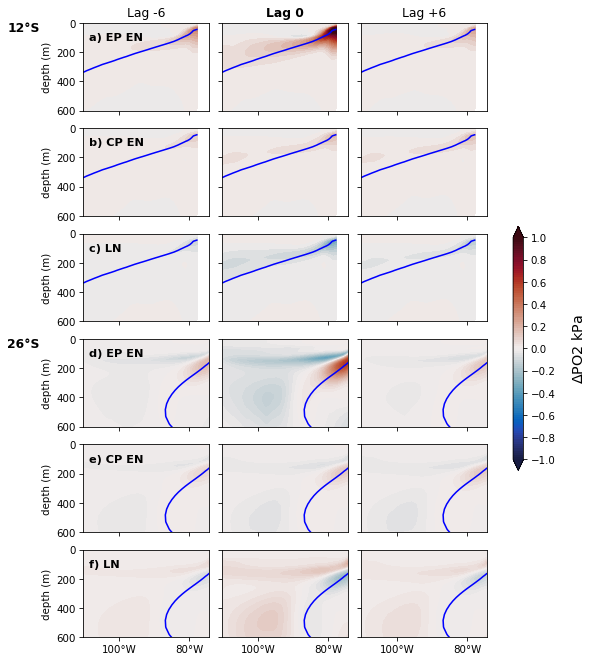


**Supplementary Figure 1: Evolution of averaged pO_2_ anomalies during EP and CP (El Niño and La Niña) events along sections (110°W-coast and 0-600m) at 12°S and 26°S.** Lag is in month and Lag 0 indicates the peak phase of the events. Anomalies are calculated as $\alpha_{PO2}\times E$ for EP EN and $\beta_{PO2}\times C$ for CP EN events and LN events. α and β stand for the regression coefficients of the anomalies onto the E and C indices at lag zero (see Methods and Supplementary Figure 3).

**
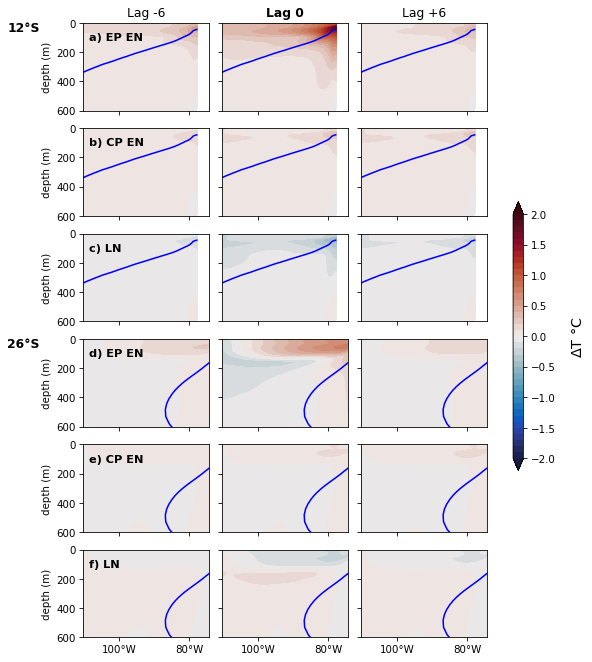
Supplementary Figure 2: Evolution of averaged temperature anomalies during EP and CP (El Niño and La Niña) events along sections (110°W-coast and 0-600m) at 12°S and 26°S.** Lag is in month and Lag 0 indicates the peak phase of the events. Anomalies are calculated as $\alpha_{T}\times E$ for EP EN and $\beta_{T}\times C$ for CP EN events and LN events. α and β stand for the regression coefficients of the anomalies onto the E and C indices at lag zero (see Methods and Supplementary Figure 3).


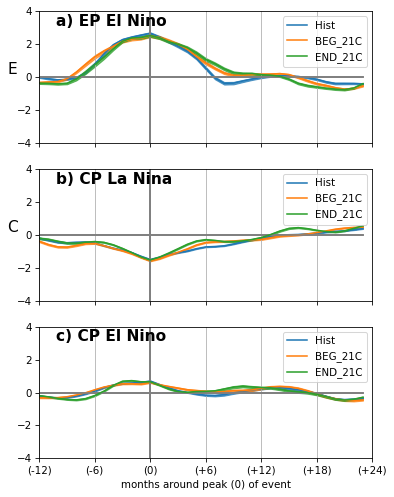


**Supplementary Figure 3: Composite evolution of the E and C indices for ENSO events.** a) EP El Niño, b) La Niña and c) CP El Niño events for three sub periods: 1920-2005 (‘HIST’), 2006-2050 (‘BEG_RCP’) and 2050-2100 (2050-2100). The envelope represents ± the standard deviation amongst 10000 composites obtained using a bootstrap method (see Method). E and C are dimensionless.


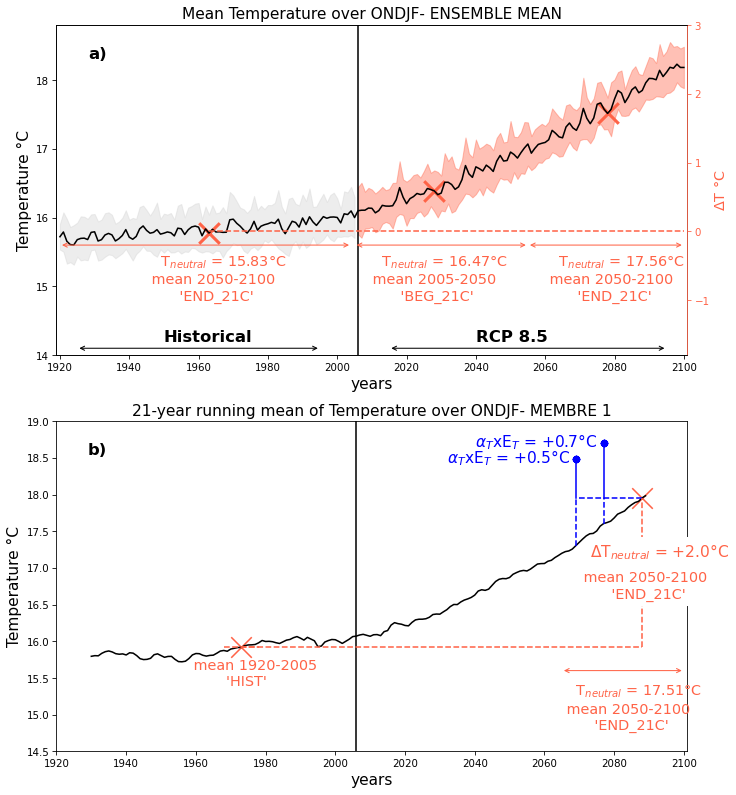


**Supplementary Figure 4: Method to calculate changes in suitable habitat during El Niño in different configurations using temperature**. a) Ensemble mean of surface temperature (black line) in the SEP in the historical (1920-2005) and RCP 8.5 (2006-2100) scenarios. The red crosses represent the middle of the three periods: 1920-2005 (‘HIST’), 2006-2050 (‘BEG_21C’) and 2050-2100 (‘END_21C’). The right y-axis represents the temperature difference relative to ‘HIST’. ΔT_neutral_ represents the mean ONDJF climatologies of each sub period. The envelope is ± the standard deviation representing the internal variability. b) Surface temperature simulated by the first member of CESM-LE. It provides a representation of the methodology used to calculate ΔVPO2_crit_. The black line is the 21-running mean (for more clarity of the figure) surface temperature of the 1^st^ member of CESM-LE. The blue dots and line represent El Niño events and their respective anomalies calculated as $\alpha\times E$. For example, ΔVPO2_crit_ in the historical period (‘HIST’) is $\Delta VPO2_{{crit}_{NINO}}=VPO2\left( PO2_{HIST}+\alpha_{PO2}\times E,T_{HIST}+\alpha_{T}\times E \right)-VPO2\left( PO2_{HIST},T_{HIST} \right)$. And the difference in VPO2_crit_ between the ‘Hist’ and ‘END_21C’ periods is: $\Delta VPO2_{crit}=VPO2\left( PO2_{END\_21C},T_{END\_21C} \right)-VPO2\left( PO2_{HIST},T_{HIST} \right)$.


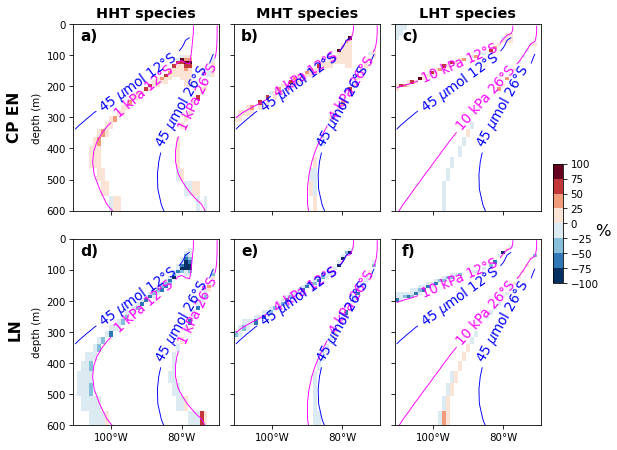


**Supplementary Figure 5: Probability density of changes in habitat suitability associated with the peak CP events (top row) and La Ni**ñ**a events (bottom row) in the present climate (1920-2005) as a function of hypoxia tolerance levels at 12°S and 26°S**. The probability density is presented as a percentage, the number of events when habitat change occurs divided by the total number of events. The red (blue) shading represents a gain (loss) of the cell, if the cell becomes metabolically suitable (unsuitable). Results at 12°S and 26°S are shown in the same panel. To locate the latitudes, the 45 µmol isocontours and the P_crit_ at T_ref_ are shown in blue and magenta lines, respectively.


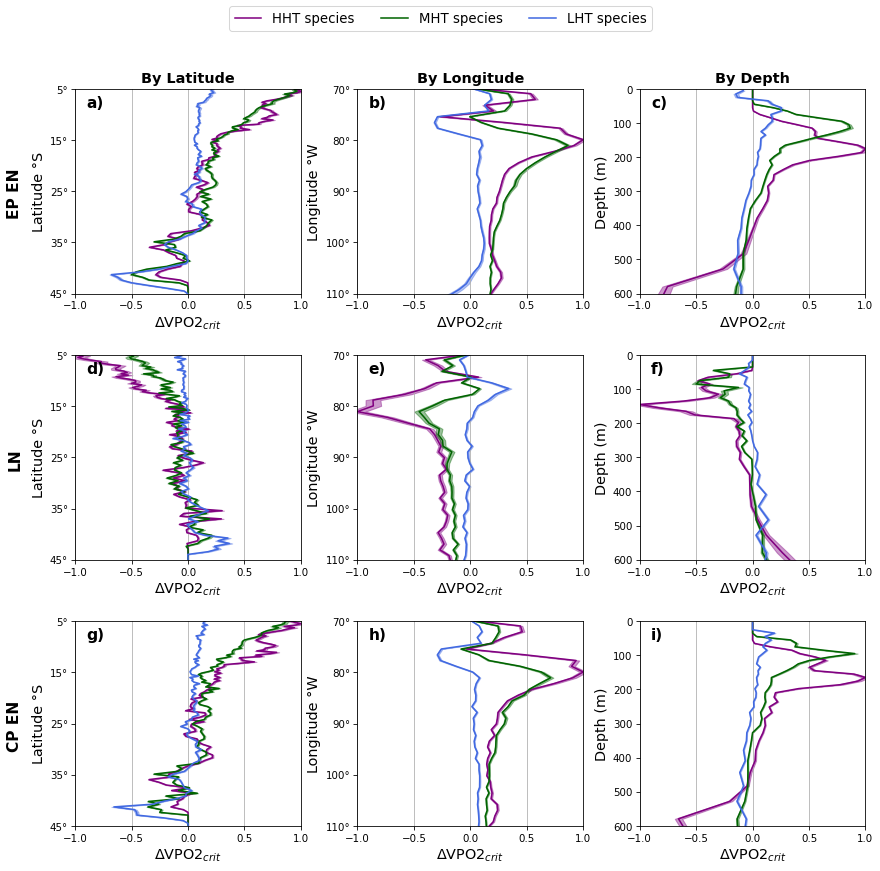


**Supplementary Figure 6: ΔVPO2_crit_ averaged over (left) latitude, (middle) longitude and (right) depth in the historical climate (1920-2005).** ΔVPO2_crit_ is calculated over the 3D domain 5-50°S, 110°W-coast and 0-600m in the historical scenario for the 3 levels of hypoxia tolerance. The envelope is ± the standard deviation amongst 10000 composites obtained using a bootstrap method. In each panel, ΔVPO2_crit_ is normalised by dividing by the maximum ΔVPO2_crit_ across species.


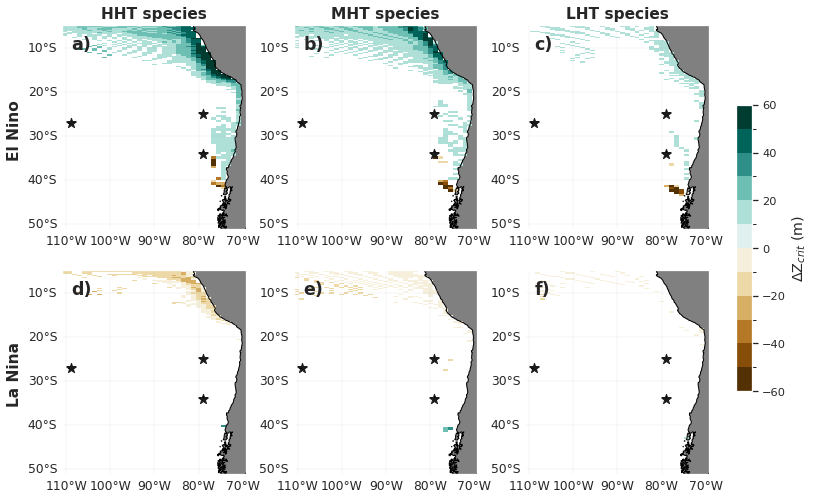


**Supplementary Figure 7: Average depth variation of Z_crit_ due to ENSO events in the upper 600m.** Positive (negative) values mean a deepening (shoaling) of the depth where pO_2_ > P_crit_. The stars represent the archipelagos of Easter Island, Juan Fernandez and Desventuradas. Z_crit_ is the depth at which pO_2_ = P_crit_.


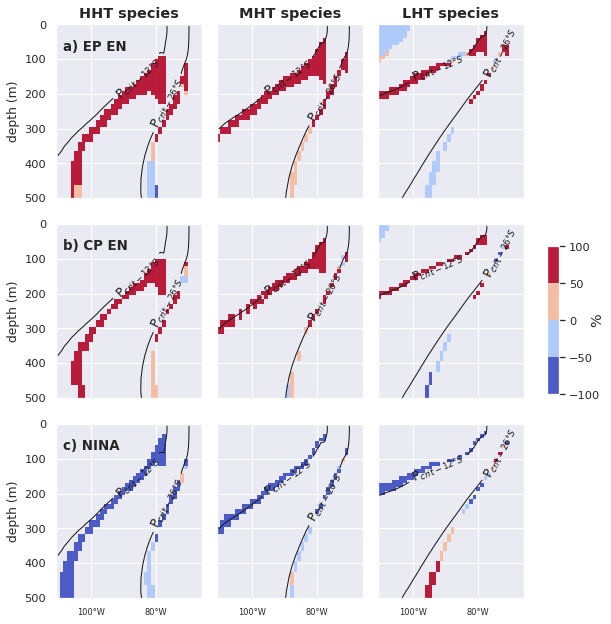


**Supplementary Figure 8: Spatial representation of the contribution of pO_2_ (ΔpO_2_) and temperature (ΔP_crit_) to changes in the balance between oxygen supply and demand during EP EN and LN along vertical sections at 12°S and 26°S in the historical climate where changes in habitat suitability occur**. Variations in the balance between oxygen supply and demand are equal to $\left| \Delta PO_{2} \right|+\left| \Delta P_{crit} \right|$ . The contribution of oxygen can then be calculated as equivalent to $\frac{\Delta PO_{2}}{\left( \left| \Delta PO_{2} \right|+\left| \Delta P_{crit} \right| \right)}$. Thus, the blue (red) shading means that oxygen contributes to reducing (increasing) ΔVPO2_crit_ during ENSO. A value > 50% means that oxygen is the main driver of ΔVPO2_crit_, conversely a value below 50% means that temperature is the main driver. The contribution is presented for MHT species (P_crit_ = 4 kPa). The thick black contours where pO_2_=P_crit_ at T_ref_ of HHT, MHT and LHT species allow to visualise what drives ΔVPO2_crit_ of each species at the limit of their habitat in neutral conditions. Cells where there are no changes in habitat suitability have been masked.

.

**
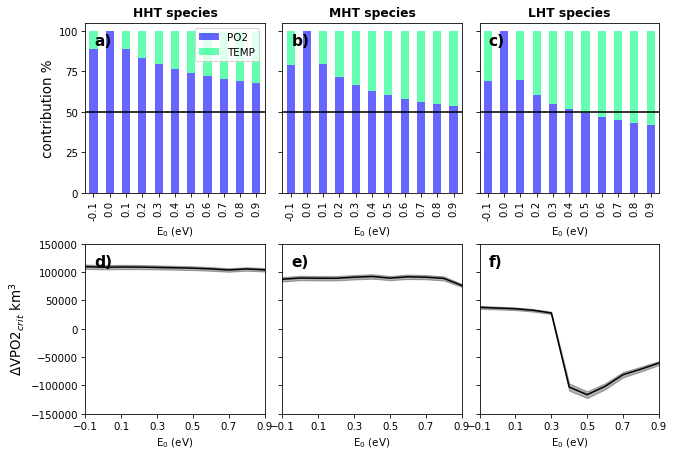
**

**Supplementary Figure 9: Effect of the temperature sensitivity parameter E_0_ during EP EN.** E_0_ varies between -0.1 to 0.9 eV as per the range documented in Deutsch et al. (2020). Panels in the top row (a, b, c) show the contribution of either oxygen (‘pO_2_’) or temperature (‘TEMP’) to the total change in volume of suitable habitat in the historical climate (‘blue’) and future climate (green). Panels in the bottom row (d, e, f) show ΔVPO2_crit_ as a function of E_0_ in the historical climate. The shading represents the standard deviation of the 10000 composites using a bootstrap method.


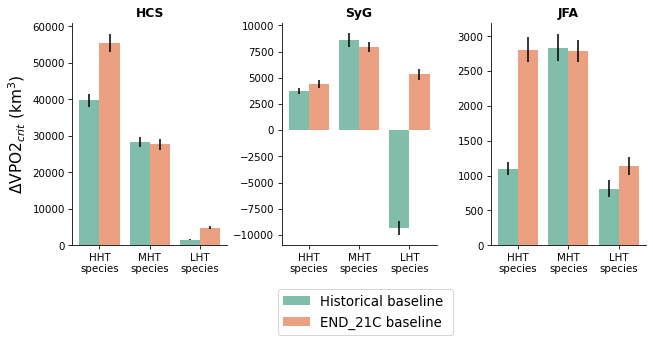


**Supplementary Figure 10: Change in habitat (ΔVPO2_crit_) due to El Niño with and without long-term warming in the future climate**. The vertical bar represents the standard deviation of the composites using a bootstrap method (see methods). (left) the Humboldt Current System (HCS), (middle) Salas y Gomez ridge (SyG) and (right) the Juan Fernandez archipelago (JFA). The red (green) bar was calculated using the mean climatology of the ‘END_21C’(Historical) periods. pO_2_ and temperature anomalies in both conditions are of the ‘END_21C’ period, hence reflecting the impact of climate change. For instance, pO_2_ during El Niño using the historical baseline is $PO2_{EN}=\underline{PO2_{{neutral}_{HIST}}}+\Delta PO2_{END\_21C}$ and pO_2_ using the END_21C baseline is $PO2_{EN}=\underline{PO2_{neutral_{END\_21C}}}+\Delta PO2_{END\_21C}$.


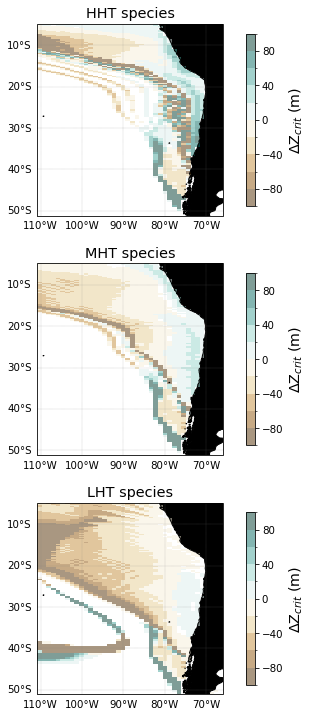


**Supplementary Figure 11: Mean depth variation of Z_crit_ (m) due to climate change**. ΔZ_crit_ is here calculated as the difference in Z_crit_ between the historical and the END_21C period (2050-2100). Positive (negative) values mean a deepening (shoaling) of the depth where pO_2_ > P_crit_. Z_crit_ is the depth at which pO_2_ = P_crit_. A ΔZ_crit_< 0 means a shoaling of Z_crit_ across climates.


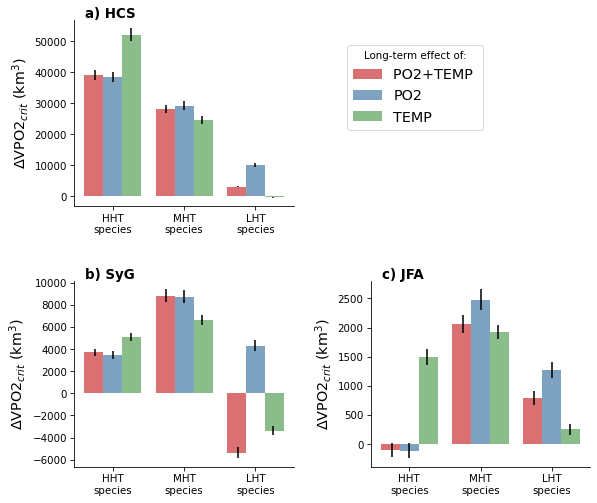


**Supplementary Figure 12. Effect of the long-term trends in pO_2_ or temperature on habitat change during El Niño in the future climate across regions and species.** The effect of pO_2_ and/or temperature is shown using mean pO_2_ and temperature (red bar), mean pO_2_ (blue bar) or mean temperature (green bar) from the END_21C period. For the last two conditions, temperature and pO_2_ respectively are from the historical climate. The anomaly due to EP EN is of the END_21C period, in all conditions. ‘BEG_21C’ and ‘END_21C’ cover 2006-2050 and 2050-2100, respectively.


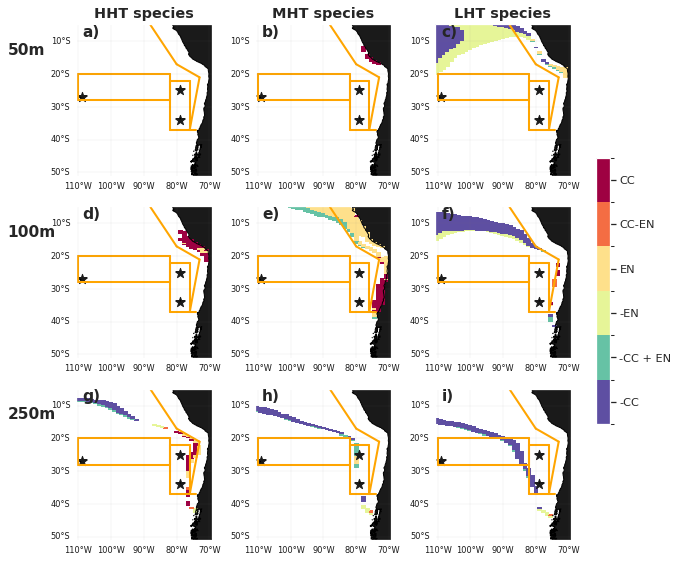


**Supplementary Figure 13. Patterns of changes in suitable habitat due to CC and/or EN.** We identify if CC or EN produce a gain or loss of suitable habitat (VPO2_crit_) in each sub-region. It leads to 6 different combinations of gain/loss due to EN and/or CC. A “+”(“-”) sign means that EN or CC cause a gain (loss) of habitat suitability. For instance, the combination “+CC -EN” means that the cell _i,j_ is gained due to climate change but is lost during EN. The orange boxes represent the subregions considered in Figure 6.
